# Leader in Global Influenza Prevention and Control Retires after 37 Years

**DOI:** 10.1111/irv.12298

**Published:** 2014-11-28

**Authors:** CDC Influenza Division Staff

*Please cite this paper as:* CDC Influenza Division Staff. Leader in Global Influenza Prevention and Control Retires after 37 Years. Influenza and Other Respiratory Viruses 8(6), 663.

At the end of November, ISIRV cofounder and *Influenza and Other Respiratory Viruses* editorial board member Dr Nancy Cox retired from her position as Director of the U.S. Centers for Disease Control and Prevention's Influenza Division. She left this position after 37 years of distinguished federal service at CDC, where she also served as the Director of one of five World Health Organization Collaborating Centers for the Surveillance, Epidemiology and Control of Influenza. Dr Cox started working on influenza at CDC in 1976. After almost four decades and 278 publications, she has transformed the surveillance and science of influenza viruses and vaccines. Her scientific work has been critical, given the tremendous global burden of human seasonal influenza – 1 billion cases, 3–5 million severe illnesses, and 300–500 000 deaths each year. In addition, avian or swine influenza viruses can adapt to human-to-human transmission, leading to global and devastating pandemics with disruption in commerce and tragic numbers of illnesses and deaths. The breadth and depth of influenza's impact is vast, underscoring Dr Cox's vital contribution from her science and service.

Dr Cox was a key founding member of ISIRV. She and the other cofounders formed the organization in 2006 and signed its original charter, establishing the core principles of scientific integrity and independence that remain the hallmarks of ISIRV policy today. ISIRV was established in part to be the home for the ‘Options for the Control of Influenza’ series of meetings. ‘Options VI’ held in Toronto, Canada in 2007 was the first meeting held under the ISIRV umbrella and was organized by Dr Cox and her CDC colleagues.

Dr Cox is an Iowa native (born in Emmetsburg) and attended Iowa State University where she received a BS in Bacteriology in 1970. She is a life member of the ISU alumni association. In addition to her Iowa State degree, she has a doctorate in virology from the University of Cambridge and was a post-doctoral fellow at both the University of Maryland, Baltimore County and at the Centers for Disease Control and Prevention.

Dr Cox has been recognized by virologists, public health officials, leaders in state and federal governments, international leaders, academics, and others. She is the recipient of multiple CDC recognition awards, including 4 CDC Shepard Awards for excellence in scientific publications, the Lancet ‘Paper of the Year,’ Time Magazine's ‘The Time 100: People Who Shape Our World,’ Service to America Award, CDC's Lifetime Achievement Award and the USG-wide award for Federal Employee of the Year. She has been a reviewer of numerous articles, the chair of many scientific panels and symposia and has served on various scientific committees for international conferences and global influenza policy development workgroups.

During her 37 years at CDC, Dr Cox has served as mentor, educator, supervisor, and supporter to hundreds of individuals: undergraduate, medical and PhD students, post-doctoral fellows, laboratory and epidemiology staff members, journalists, and visiting researchers. As Director of the WHO Collaborating Center for the Surveillance, Epidemiology and Control of Influenza at CDC, Dr Cox has worked closely with public health laboratory officials from Russia, Vietnam, and China, leading to transforming their capabilities in influenza virology and surveillance. Her oversight of influenza laboratories at CDC has set the standards for measuring immune response in infected and vaccinated people and also has led CDC to be the global reference center for antiviral resistance and for measuring transmission of influenza viruses in animal models. Her leadership at WHO has also led to significant changes in the methods, reporting, interpretation, and policy development for selecting candidate vaccine viruses for use in annual vaccine production. Through Dr Cox's scientific rigor, knowledge and foresight, she leaves a legacy of improved capacity for the study of influenza virus across the globe.

